# Spontaneous Heterotopic Pregnancy: Diagnosis and Management

**DOI:** 10.1155/2022/2994808

**Published:** 2022-07-26

**Authors:** Katie P. Nguyen, Marlekah Hudspeth, Honey Milestone

**Affiliations:** ^1^Department of Family Medicine, Riverside Community Hospital, HCA Healthcare, Riverside, CA, USA; ^2^University of California Riverside, School of Medicine, Riverside, CA, USA; ^3^Department of Obstetrics & Gynecology, University of California Riverside, School of Medicine, Riverside Community Hospital, Riverside, CA, USA

## Abstract

**Background:**

Heterotopic pregnancies albeit rare are conceivably life-threatening if missed. With the development of assisted reproductive techniques, the incidence has increased. Confirmation of an intrauterine pregnancy (IUP) should not preclude the existence of a heterotopic pregnancy.

**Case:**

A healthy 27-year-old patient (gravida 4, term 1, preterm 0, abortion 2, living 1) at approximately 5 weeks gestation through natural conception presented to the emergency room with acute abdominal pain and vaginal bleeding. Pelvic ultrasound showed evidence of an IUP and a right adnexal mass, raising suspicion for a heterotopic pregnancy. The patient underwent an uncomplicated laparoscopic right salpingectomy. An IUP was confirmed on ultrasound postoperatively. The patient had an early pregnancy loss at 8 weeks of gestation.

**Conclusion:**

With a high index of suspicion from clinical presentation and pelvic imaging, heterotopic pregnancy, while rare, should not be ruled out.

## 1. Introduction

A heterotopic pregnancy is the presence of an intrauterine pregnancy (IUP) and a concurrent extrauterine pregnancy. It is both rare and life-threatening. The risk of having a heterotopic pregnancy from natural conception is estimated to be from 1 in 4,000 to 1 in 30,000 [1]. With the emergence of assisted reproductive technology, the incidence has been reported to be as high as 1 in 100 [1, 2]. Despite its rarity, the presence of a confirmed IUP should not exclude a concurrent extrauterine pregnancy. Ectopic pregnancies and heterotopic pregnancies are similar in presentation. Obtaining serial human chorionic gonadotropin (hCG) has limited diagnostic value as production is likely from the intrauterine pregnancy. This case report details the diagnosis and management of a naturally conceived heterotopic pregnancy in a healthy patient.

## 2. Case Presentation

**Figure 1:**
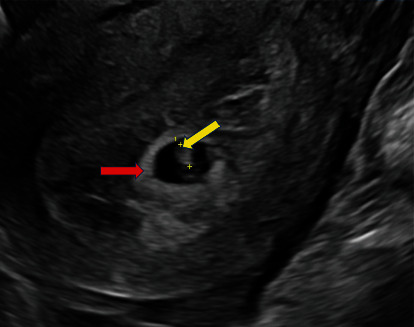
Longitudinal transvaginal ultrasound demonstrates an intrauterine gestational sac (red arrow) with a fetal pole (yellow arrow). Crown rump length measures 0.37 cm which correlates to the gestational age of 5 weeks and 5 days. Fetal heart rate was 128 beats per minute.

**Figure 2:**
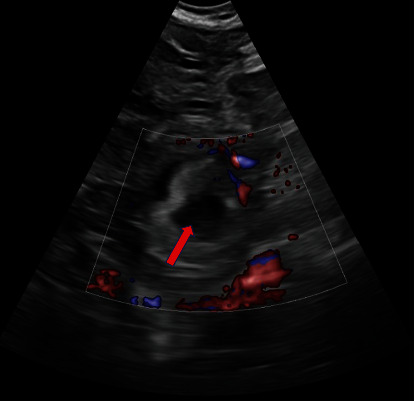
Longitudinal transvaginal ultrasound of the right adnexa demonstrates a thick walled hypoechoic cystic structure (red arrow) with weak peripheral vascularity (“ring of fire”). There is no fetal pole present.

**Figure 3:**
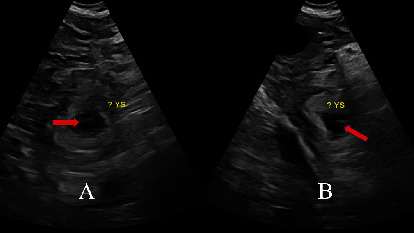
Longitudinal (a) and transverse (b) transvaginal ultrasound images of the right adnexa structure demonstrate possible internal echogenicity or septations (red arrow) which may represent a yolk sac or reverberation artifact.

**Figure 4:**
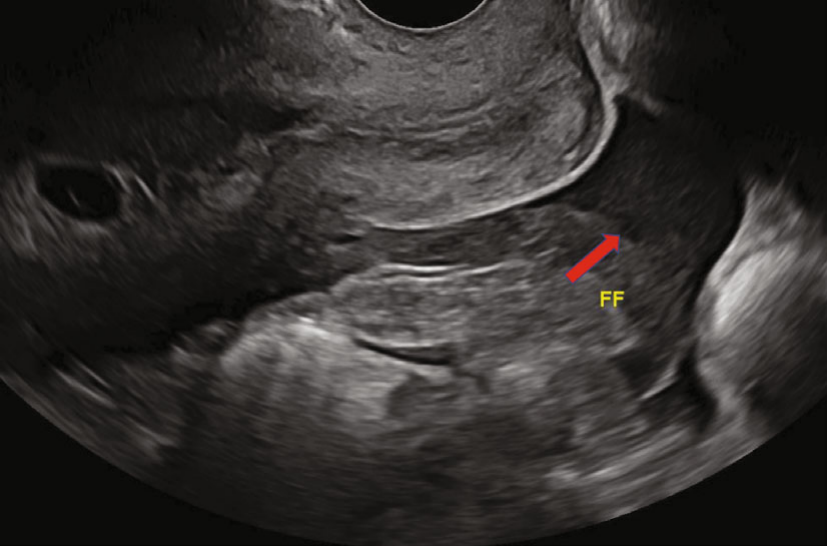
Sagittal transvaginal ultrasound image of the cervix with the posterior cul-de-sac with free fluid concerning for hemorrhage (red arrow).

A healthy 27-year-old patient (gravida 4, term 1, preterm 0, abortion 2, living 1) at 5 weeks and 5 days gestation through natural conception presented to the emergency department with acute lower abdominal pain and vaginal bleeding. She had associated nonbilious nonbloody vomiting. Obstetrical history included one spontaneous vaginal delivery and two spontaneous abortions. The medical, surgical, and family histories were noncontributory. On presentation, she was tachycardic at 117 beats per minute. The physical exam was significant for rebound tenderness of the lower abdomen, worse on the right side at McBurney's point. The pelvic exam showed blood in the vaginal vault. Labs were notable for an hCG at 16,108 mIU/mL and leukocytosis. Pelvic ultrasound showed evidence of an IUP measuring 5 weeks and 5 days with a fetal heart rate of 128 bpm (Figure 1). A 2.4 cm hyperechoic structure with a 1.3 cm internal hypoechoic structure was noted in the right adnexa. Weak peripheral vascularity was noted. No fetal pole was identified (Figure 2). There was a weak “ring of fire” sign present with internal septations possibly indicative of a yolk sac (Figure 3). There was a small volume of complex free fluid in the pelvis (Figure 4). Vascular waveforms were seen in both ovaries.

**Figure 5:**
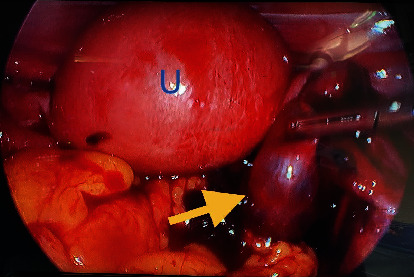
Intraoperative view of an enlarged uterus (U) and enlarged right fallopian tube with a purple-red hue (yellow arrow).

**Figure 6:**
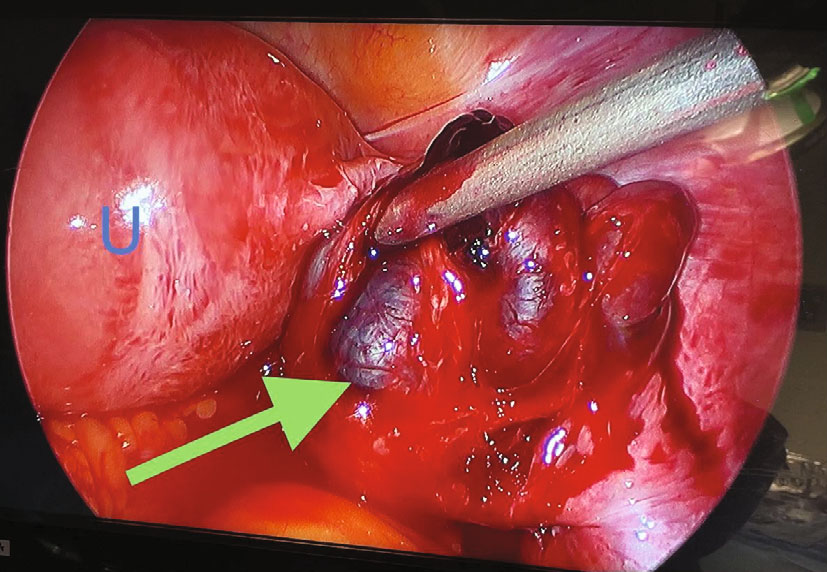
Intraoperative view of an enlarged uterus (U) and enlarged right fallopian tube with hemoperitoneum from the ruptured ectopic gestation (green arrow).

**Figure 7:**
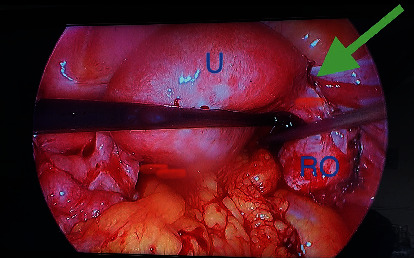
Intraoperative view of an enlarged uterus (U), right ovary (RO), and right salpingectomy (green arrow).

Given findings of an acute abdomen and pelvic ultrasound highly suspicious for an ectopic pregnancy, the patient was consented for laparoscopic evaluation. The patient was counseled on the risk of having an early pregnancy loss with the surgical intervention. General surgery was consulted for possible appendectomy. The patient underwent a laparoscopic right salpingectomy with evacuation of hemoperitoneum. Significant operative findings included an enlarged uterus, an enlarged right fallopian tube with a purple-red hue, and notable rupture with hemoperitoneum (Figures 5, 6, and 7). The estimated blood loss was 500 mL. Bilateral ovaries, left fallopian tube, appendix, and liver were normal in appearance. The pathology results confirmed the presence of the right fallopian tube from the ruptured ectopic gestation. The postoperative course was unremarkable. A postoperative ultrasound confirmed a viable IUP, and the patient was discharged home on postoperative day one. Follow up confirmed an early pregnancy loss at 8 weeks of gestation.

## 3. Discussion

Heterotopic pregnancies are rare, but could be life-threatening if missed. The presence of a confirmed IUP usually rules out an extrauterine pregnancy if there is no evidence for another concurrent pregnancy located elsewhere. A systematic review of the literature from 2005 to 2010 revealed that as many as 33% of heterotopic cases had prior sonography of a normal IUP which led to false reassurance and misdiagnosis [3]. Studies report a wide variability for hCG concentrations in early pregnancy warranting cautious interpretation of a single hCG value for the diagnosis and management of suspected ectopic and/or heterotopic pregnancies. At around 6 weeks gestation, the hCG range could vary from 105 to 106,520 mIU/mL [4, 5]. A review of the literature shows a wide range of hCG levels for heterotopic pregnancies at similar gestational ages [6, 7]. With in vitro fertilization studies, hCG levels tend to be lower in heterotopic pregnancies compared to intrauterine twin pregnancies, but tend to be higher than intrauterine single pregnancies [8]. Patients who have higher than expected hCG levels especially with known singleton gestations need close monitoring with repeat hCG levels and ultrasound to subsequently rule out multiple gestations and heterotopic pregnancies. Prompt and accurate diagnosis remains a challenge. Major risk factors include prior tubal diseases or use of ART. There are also heterotopic pregnancy cases without any risk factors as demonstrated by the patient in this case report. The most common presentation is abdominal pain and vaginal bleeding which should prompt evaluation for an ectopic pregnancy versus a threatened abortion. In fact, a presumptive diagnosis of heterotopic pregnancy can be made if a patient has a combination of abdominal pain, signs of peritonitis, and ultrasound findings showing an adnexal mass with an enlarged uterus. There are also heterotopic pregnancies that are asymptomatic [3].

The diagnosis of heterotopic pregnancy is straightforward with the sonographic visualization of both an IUP; a complex adnexal mass containing either a yolk sac, embryo, or fetal pole; or echogenic fluid in the posterior cul-de-sac [9]. The difficulty lies in differentiating between an adnexal mass with a gestational sac but no embryo present versus a hemorrhagic corpus luteal cyst [10]. Doppler ultrasound allows assessment of the maternal fetal circulation and the uteroplacental blood flow velocity waveform; there is continuous maternal blood flow in the intervillous space, and the fetal blood flow velocity waveform is detectable as early as 5 weeks of gestation [9, 11]. Transvaginal color Doppler ultrasound can detect a high diastolic vascular flow, also known as the “ring of fire” sign, for a gestational sac of abnormal location if the ectopic pregnancy is unruptured [12]. Echogenic free fluid, if present, is suggestive of intraperitoneal hemorrhage from a ruptured ectopic pregnancy as was the case for this patient. If there was prior ovarian hyperstimulation, enlarged ovaries would be present [12].

This patient had evidence of an IUP measuring at 5 weeks with cardiac activity (Figure 1). There was a right adnexal hypoechoic structure with an internal hypoechoic structure. The “ring of fire” sign is indicative of a hypervascular lesion with peripheral vascularity commonly seen in corpus luteal cysts or an ectopic pregnancy. For every IUP, its corpus luteal cyst with the “ring of fire” is located in the ovary and not in the adnexa. The diagnosis was not definitive here because despite its presence in the adnexa, the “ring of fire” was weak and without a fetal pole. The possible internal echogenicity or septations seen may represent a yolk sac or artifact (Figures 2 and 3). With a high index of suspicion from the clinical presentation (pregnant patient with acute abdomen and vaginal bleeding) and concerning pelvic ultrasound (i.e., IUP, free fluid in pelvis, and complex adnexal mass), the possibility for heterotopic pregnancy, albeit rare, cannot be ruled out (Figure 4).

If clinical presentation and/or sonography findings are not definitive as was the case for this patient, a diagnostic laparoscopy or laparotomy (if hemodynamically unstable) would be indicated. This patient's serial abdominal exams worsened which warranted prompt surgical evaluation (Figures 5, 6, and 7). Appropriate management of heterotopic pregnancies requires taking into consideration the following: hemodynamic stability, prognosis or desired outcome for the IUP, site of implantation of the ectopic pregnancy, and the least invasive therapeutic approach. Laparoscopy is preferred over laparotomy if the patient is hemodynamically stable. Patients who are hemodynamically unstable are more likely to require laparotomies to prevent hemoperitoneum and hypovolemic shock from a ruptured ectopic pregnancy. The first line of treatment is a salpingectomy. Extra precautions should be taken to avoid cannulation or excessive manipulation of the uterus in order to preserve the IUP [13]. If the IUP is not desired, patients who are hemodynamically stable with definitive diagnosis on ultrasound are candidates for conservative systemic medical management with methotrexate. If the IUP is desired and the ectopic pregnancy is unruptured, consider transvaginal local injection of feticidal substances such as potassium chloride or hyperosmolar glucose. Both are effective in terminating the ectopic pregnancy, do not affect the fallopian tube, and are tolerated by the IUP [14]. Patients should still be counseled that conservative management could fail and that subsequent salpingectomy may be required. While it is crucial to counsel the patient on the increased risk of the IUP ending in an early pregnancy loss, a review of the literature shows an improvement in the survival rate from between 48% and 51% in 1957 [15] to 69% in 2007 [3].

Having a high index of suspicion for heterotopic pregnancy, despite its rarity, allowed prompt diagnosis and management in this otherwise healthy pregnant patient with natural conception.

## Data Availability

The data that support the conclusions of the study are available from the corresponding authors upon reasonable request.
